# Predictors of Indoor Air Concentrations in Smoking and Non-Smoking Residences

**DOI:** 10.3390/ijerph7083080

**Published:** 2010-07-04

**Authors:** Marie-Eve Héroux, Nina Clark, Keith Van Ryswyk, Ranjeeta Mallick, Nicolas L. Gilbert, Ian Harrison, Kathleen Rispler, Daniel Wang, Angelos Anastassopoulos, Mireille Guay, Morgan MacNeill, Amanda J. Wheeler

**Affiliations:** 1 Health Canada, 269 Laurier Avenue West, Ottawa, Ontario K1A 0K9, Canada; E-Mails: Nina.Clark@hc-sc.gc.ca (N.C.); Keith.Van.Ryswyk@hc-sc.gc.ca (K.V.R.); Ranjeeta.Mallick@hc-sc.gc.ca (R.M.); Nicolas.Gilbert@hc-sc.gc.ca (N.L.G.); Mireille.Guay@hc-sc.gc.ca (M.G.); Morgan.MacNeill@hc-sc.gc.ca (M.M.); Amanda.Wheeler@hc-sc.gc.ca (A.W.); 2 Regina Qu’Appelle Health Region, 2110 Hamilton Street, Regina, Saskatchewan S4P 2E3, Canada; E-Mails: Ian.Harrison@rqhealth.ca (I.H.); Kathleen.Rispler@rqhealth.ca (K.R.); 3 Environment Canada, 335 River Road, Ottawa, Ontario K1A 0H3, Canada; E-Mail: Daniel.Wang@ec.gc.ca; 4 Carleton University, 1125 Colonel By Drive, Ottawa, Ontario K1S 5B6, Canada; E-Mail: angelos@cogeco.ca

**Keywords:** residential indoor air quality, exposure, sources

## Abstract

Indoor concentrations of air pollutants (benzene, toluene, formaldehyde, acetaldehyde, acrolein, nitrogen dioxide, particulate matter, elemental carbon and ozone) were measured in residences in Regina, Saskatchewan, Canada. Data were collected in 106 homes in winter and 111 homes in summer of 2007, with 71 homes participating in both seasons. In addition, data for relative humidity, temperature, air exchange rates, housing characteristics and occupants’ activities during sampling were collected. Multiple linear regression analysis was used to construct season-specific models for the air pollutants. Where smoking was a major contributor to indoor concentrations, separate models were constructed for all homes and for those homes with no cigarette smoke exposure. The housing characteristics and occupants’ activities investigated in this study explained between 11% and 53% of the variability in indoor air pollutant concentrations, with ventilation, age of home and attached garage being important predictors for many pollutants.

## 1. Introduction

Residential indoor air quality is increasingly recognized as an important determinant of health, being associated with both acute and chronic health outcomes. Indoor air pollutants, either infiltrating from the outside or produced by indoor sources, have been linked with a wide range of health effects, including asthma and allergy symptoms, airway irritation, decreased lung function and other respiratory symptoms [1–3]. Furthermore, several time-activity surveys have reported that individuals spend, on average, two-thirds of their time inside their homes [4,5]. As a result, personal exposure to indoor airborne pollutants can be greatly impacted by concentrations found in residential environments [3,6].

Canada is one of the few countries to have adopted guidelines specifically for residential indoor air. In 1987, Health Canada, the Canadian federal department of health, published the *Exposure Guidelines for Residential Indoor Air Quality* through the *Federal-Provincial Advisory Committee on Environmental and Occupational Health* [7]. However, given the wealth of science that has emerged since this time, these guidelines are currently being reviewed to better protect Canadians from potential health risks in their homes.

When new exposure limits are proposed for indoor air pollutants, it is important to be able to compare them with levels currently found in homes. In order to do so, Health Canada is collecting extensive data for a range of indoor air pollutants typically found in residences, including both particle- and gas-phase pollutants. The current study in Regina was undertaken as part of a series of indoor air quality studies that have been conducted across Canada. The main objectives of these studies were to determine the concentrations of indoor air pollutants in Canadian homes; to determine the association between these pollutants and Canadian housing characteristics; and to assess the impact that occupant activities have upon concentrations. This information is needed to develop the science of indoor air quality further, and guide the development of appropriate actions to improve indoor air quality, including the review and update of Health Canada’s *Residential Indoor Air Quality Guidelines*.

## 2. Experimental Section

### 2.1. Study Design

In 2007 Health Canada, in collaboration with *Regina Qu’Appelle Health Region* (RQHR), conducted residential indoor and outdoor measurements in Regina, Saskatchewan, Canada for a range of air pollutants. The air pollutants measured included: fine and coarse size fractions of particulate matter (PM_2.5_ and PM_10-2.5_), elemental and organic carbon (EC/OC), nitrogen dioxide (NO_2_), ozone (O_3_), carbon monoxide (CO), individual volatile organic compounds (VOCs), and aldehydes (formaldehyde, acetaldehyde, and acrolein). Ancillary measurements included air exchange rates, temperature and relative humidity. One settled dust sample was also collected in each home, and analyzed for common allergens. A subset of the indoor air pollution data is presented here.

**Figure 1.**
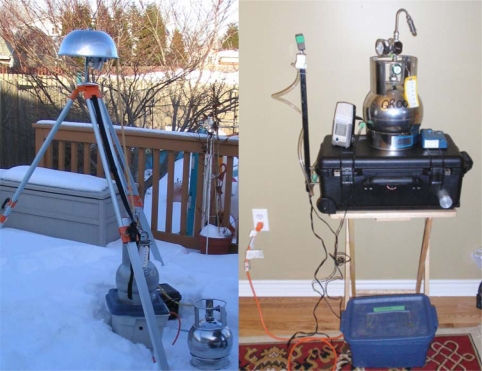
Examples of outdoor and indoor experimental set-ups in Regina.

Indoor measurements were typically taken at breathing height (1.5 m) within the family or living room where participants spent a substantial amount of time (see Figure 1). All air pollutants, except carbon monoxide and the aldehydes, were also measured outdoors in the participants’ backyard, as far away from the home as possible and away from any combustion sources such as barbecues and driveways. The air pollution measurements were conducted in both the winter (January to March) and summer (July and August) seasons. There were 10 sampling weeks per season, with 12 homes measured concurrently per week. Depending on the pollutant, measurements were conducted during the first 24 hours (h), or for an integrated five day period, or both. Participants were asked to continue with their normal activities throughout the sampling period in order to ensure that the concentrations measured were representative of typical residential exposures. The dust sample was collected on a separate visit, since participants were asked not to vacuum for seven days prior.

Participants were selected from a list of respondents living within the city limits who agreed to participate using an initial randomized telephone survey conducted by RQHR. The residences were selected based on the *a priori* hypotheses that age of the home as well as heating and cooking appliances would impact indoor air quality; as such, sampling was stratified by age of home and the residences were grouped into five subsets of construction year (1945 and before, 1946–1960, 1961–1980, 1981–2000, and 2001–2006) and presence of gas stoves.

Approval was obtained from Health Canada’s Research Ethics Board to conduct this study. All personal information was protected according to the Canadian *Access to Information Act* and the *Privacy Act*. Informed consent was obtained from the participants on the first visit to their home, prior to the administration of questionnaires and the set-up of the equipment. At the end of the study, participants were provided with a personalized report describing the data collected in their home in comparison to Canadian guidelines and standards, as well as to the other homes that were monitored during the same week. The report also contained guidance material from Health Canada and the Canadian Mortgage and Housing Corporation (CMHC) on how to reduce their exposure to air pollutants inside their home.

### 2.2. Questionnaires

Participants completed two different questionnaires to identify potential sources of indoor exposure and factors that may influence concentrations of air pollutants. An interviewer-administered baseline questionnaire was completed to obtain information on housing characteristics that would not change over the course of the sampling period, such as: age and type of home, dimensions, heating and cooking systems, recent addition of furniture, recent painting or varnishing, recent flooding and presence of mould. During the second sampling season, a shorter baseline questionnaire was administered to returning participants to assess any new renovations and changes that may have occurred in the residence since the last season of sampling.

A daily participant-administered questionnaire was completed to obtain information on daily activities in the home during the sampling period such as: cleaning and cooking, use of personal care products, opening and closing of windows, presence of pets and smokers in the home. Data from the questionnaires were independently entered twice in an electronic database and then compared to identify any discrepancies in the data entry.

### 2.3. Passive Samplers

Ogawa passive samplers (Ogawa & Co., Pompano Beach, FL, USA) were used to sample for NO_2_ and O_3_. In both seasons, O_3_ was sampled for a five-day period, whereas NO_2_ was sampled for a 24-h period. Outdoor O_3_ and NO_2_ were not measured in the winter, due to temperature limitations of the sampling method. The samplers for NO_2_ were two-sided, allowing for duplicate measurements at all locations. Concentrations were measured independently for the two filters and the average concentrations were reported. Ogawa badges were analyzed by ion chromatography, according to the Ogawa Standard Operating Procedures (SOP).

Concentrations of aldehydes (formaldehyde, acetaldehyde, acrolein) were measured indoors only for 24-h per season using SKC UMEX 100 Passive Samplers (SKC Inc. Eighty Four, PA, USA), with dinitrophenylhydrazine (DNPH) as the reagent. The samples were analyzed by high performance liquid chromatography (HPLC) using US EPA Compendium Method TO-11A.

Individual volatile organic compounds (VOC) in air were collected using clean and evacuated Summa™ canisters. VOCs were collected indoors and outdoors using six liter canisters and pre-calibrated flow controllers which operated at a flow rate of 0.7 ml/min and 3.5 ml/min for five-day and 24-h sampling, respectively. The air samples were analyzed using gas chromatography-mass spectrometry (GC/MS), according to US EPA method TO-15.

### 2.4. Active Samplers

Integrated five-day samples of two size fractions of particulate matter (PM_2.5_ and PM_10-2.5_) were collected indoors and outdoors for both seasons using Harvard Coarse Impactors (HCI, Harvard School of Public Health, Boston, MA, USA). HCIs operated at a flow rate of five liters per minute (LPM) using a BGI pump (5.2 LPM Model, BGI Inc., Waltham MA, USA). Gravimetric analyses were conducted using the method outlined in the Quality Assurance Guidance Document 2.12 by the US EPA [8].

Elemental carbon and organic carbon (EC/OC) were collected indoors and outdoors during the summer season only using a ChemComb (Model 3500, Thermo Scientific, Waltham, MA, USA). ChemComb samplers operated at a flow rate of 10 LPM with a PM_2.5_ inlet using a BGI pump (10 LPM Model, BGI Inc., Waltham, MA, USA). There was a pre-fired quartz fiber filter inside the ChemComb to collect 24-h integrated samples. After exposure, the sampled filter was analyzed for carbon content using DRI Model 2001 thermal/dual-optical carbon analyzer (Atmoslytic Inc Calabasas, CA, USA) and the IMPROVE (Interagency Monitoring of Protected Visual Environments) analysis protocol.

### 2.5. Continuous Measurements

Continuous measurements of carbon monoxide (CO), temperature and relative humidity (RH) were collected indoors at each residence for five days in each season. Real-time PM_2.5_ concentrations were measured indoors and outdoors using a DustTrak (Model 8520, TSI Incorporated, Shoreview, MN, USA), which is a laser-photometer that uses light scattering technology to determine mass concentrations in real-time. The DustTrak instruments operated at a flow rate of 1.7 LPM with a time interval of one minute. Real-time CO concentrations were also measured at an interval of one minute, using a Langan CO Measurer (Model T-15n, Langan Products, San Francisco, CA, USA). Data for these continuous methods are not presented as part of this paper. Temperature and RH were recorded at 10 minute intervals using an ACR SmartReader Two data logger (ACR Systems, Inc).

### 2.6. Air Exchange Rate

Home air infiltration rates (m^3^/h) were determined for the corresponding 24-h or five-day period by the perfluorocarbon tracer (PFT) technique [9]. These samples were taken on the same floor that the indoor air measurements were made. After exposure, the capillary absorption tubes (CATs) were shipped to the laboratory and analyzed by gas chromatography with an electron capture detector (GC/ECD). For each home, the air exchange rate, expressed as air changes per hour (ACH), was calculated by dividing the infiltration rate by the estimated house volume. Due to logistical issues, air exchange rates were only available in the winter season.

### 2.7. Fixed-Site Monitors

The same monitoring instruments that were located outdoors at the homes were also collocated with the National Air Pollution Surveillance (NAPS) site instruments operated by Environment Canada at their downtown Regina location. These comparisons were conducted to assess agreement between the methods. This manuscript reports the data for PM_2.5_ and NO_2_.

### 2.8. Quality Assurance

Laboratory detection limits (LDL) were estimated as three times the standard deviation of the laboratory blanks, with field detection limits (FDL) being defined as three times the standard deviation of the field blanks. Field blanks comprised approximately 10% of all samples.

The quality assurance program included the calibration of flow rates, leak tests, collection of routine field blanks and determination of accuracy for the chemical analyses.

Blank corrections were applied when more than 50% of the field blanks were greater than the LDL. In these situations, a FDL was then calculated as being three times the standard deviation of the field blanks. Sample data were then adjusted by subtracting the median of the field blanks. Any resulting values that were lower than the LDL were substituted with half of the LDL. Samples that were above the LDL but below the FDL were unchanged.

All data were assessed for validity using the following criteria. Any samples requiring a specific flow rate were tested at the beginning and end of each sampling period; if the end flow rate was operating at more than 20% above or below the target flow value, the sample was deemed invalid. Samples were also deemed invalid if they were deployed for more or less than 20% of the time.

For the situations where more than 50% of the data were below the FDL, the descriptive statistics are presented but no models have been developed.

### 2.9. Data Analysis

All analyses were conducted using SAS v.9.1 (SAS Institute Inc., NC, USA). Data from standardized methods used at the NAPS station were used to yield estimates of bias for the few pollutants where NAPS methods were available (PM_2.5_ and NO_2_). The following bias definition, also frequently referred to as the fractional or percent difference, was utilized:

$$\text{Bias}=\frac{A-T}{T}$$

where A is the instrument value and T is the true value. This returns a positive or negative number, which can be multiplied by 100 to produce a “percent bias” which is normally reported.

Bivariate regressions between each variable and pollutant across homes were examined for each season. Distributions of indoor pollutant levels were positively skewed and were log-normally transformed for all regression analyses. Variables that were hypothesized *a priori* to influence pollutant levels were examined using plots and simple linear regression. Predictors that were found to be marginally significant (p < 0.10) in the bivariate analyses were considered for inclusion in a stepwise regression model (p < 0.10 to enter and p < 0.10 to remain) to construct a final, season-specific model for each of the pollutants by season. Diagnostic plots and Cook’s distance were examined to detect violations of the assumptions of linear modeling and presence of highly influential points. Where a relationship with a predictor was completely dependent on a highly influential point, that predictor was removed from the model. Where smoking was a major contributor to indoor concentrations, separate models were constructed for all homes and for those homes with no cigarette smoke exposure.

## 3. Results and Discussion

### 3.1. Participant Characteristics

Baseline characteristics and daily activities influencing indoor air pollutant levels are presented in Table 1. Activity statistics are for the first 24 h of monitoring, during which all pollutants were measured, with the exception of PM_2.5_ which was measured over five days. There were 106 and 111 homes sampled in winter and summer seasons, respectively, with repeated measurements completed for both seasons in 71 homes, for a total of 145 homes. All homes were single detached or row homes; there were no apartment buildings or condo units. Despite recruitment efforts, there were only six and nine homes built after 2000 for winter and summer, respectively. There were fewer homes with gas stoves in the winter than in the summer (n = 7 *vs.* 36), due to recruitment directly targeting homeowners with gas stoves during summer. There were 21 and 15 homes with smokers present in the winter and summer, respectively, resulting in the need to analyze some of the pollutants for all homes and non-smoking homes separately, in an effort to identify sources of exposure besides cigarette smoke.

### 3.2. Air Pollutant Measurements

Descriptive statistics for indoor (all homes and non-smoking homes) and outdoor air pollutants for both seasons are presented in Table 2. Due to problems with the analysis and quality control results, the winter data for NO_2_ and O_3_ were deemed invalid, and are therefore not presented. Furthermore, winter outdoor five-day VOCs samples were problematic due to the extreme cold weather, and therefore only 24-h concentrations are reported.

Indoor concentrations were above the FDL for the majority of the indoor air pollutants. More than 50% of the samples were below the respective FDL for indoor measures of acrolein (both seasons), EC (summer only) and ozone (summer only). For these pollutants, descriptive statistics are presented, but predictive models were not developed.

Significant seasonal differences in indoor concentrations were seen with higher geometric mean concentrations in summer compared to winter for formaldehyde (31.1 μg/m^3^ *vs.* 23.4 μg/m^3^, p < 0.001) and acrolein (1.1 μg/m^3^ *vs.* 0.6 μg/m^3^, p < 0.001). Outdoor geometric mean concentrations were significantly lower in the summer than in the winter for benzene (0.3 μg/m^3^ *vs.* 0.7 μg/m^3^, p < 0.001) and were higher in the summer than in the winter for PM_2.5_ (7.3 μg/m^3^ *vs.* 6.2 μg/m^3^, p = 0.004). All other pollutants did not vary significantly by season.

Geometric mean concentrations were consistently higher indoors than outdoors for benzene (1.3 μg/m^3^ *vs.* 0.3 μg/m^3^; p < 0.0001 in summer, and 1.4 μg/m^3^ *vs.* 0.7 μg/m^3^; p < 0.0001 in winter) and toluene (11.3 μg/m^3^ *vs.* 1.0 μg/m^3^; p < 0.0001 in summer, and 8.4 μg/m^3^ *vs.* 1.1 μg/m^3^; p < 0.0012 in winter). In the summer, the geometric mean concentration for ozone was higher outdoors than indoors (10.3 μg/m^3^ *vs.* 0.12 μg/m^3^; p < 0.0001); while NO_2_ was higher indoors than outdoors (8.5 μg/m^3^ *vs.* 5.4 μg/m^3^; p < 0.0001). For PM_2.5_, winter outdoor geometric mean concentrations were higher than indoors (6.2 μg/m^3^ *vs.* 5.5 μg/m^3^; p < 0.04), while no difference was seen in the summer. Indoor and outdoor concentrations for EC in the summer were not statistically different.

Smoking homes had higher PM_2.5_ levels than non-smoking homes (geometric mean = 22.0 *vs.* 5.3 μg/m^3^; p < 0.0001 for summer, and 16.7 *vs.* 4.1 μg/m^3^; p < 0.0001 for winter; data not shown). NO_2_ concentrations were higher in homes with gas stoves compared to homes with electric stoves (geometric mean 14.8 *vs.* 6.4 μg/m^3^ for summer; p < 0.0001; data not shown).

Air exchange rates were measured for the first 24 h of sampling in homes, and are only available for the winter sampling season. Air exchange rates ranged from 0.02 to 3.09 ACH, with a mean ± SD = 0.39 ± 0.38 ACH.

We further compared concentrations measured as part of this study to Health Canada’s residential indoor air quality guidelines, when available. There are currently no guidelines for benzene, toluene, acetaldehyde and acrolein in Canada. For ozone, all homes were below the 8-h exposure limit of 40 μg/m^3^ [10]. For the other pollutants, comparisons of concentrations measured in Regina with the guidelines are presented below.

### 3.3. Data Quality

The estimated biases for NO_2_ and PM_2.5_, as compared to the NAPS site data, are as follows. The 24-h NO_2_ level for summer sampling at the central NAPS site was (mean ± SD) 13.57 ± 6.15 μg/m^3^ for the Ogawa badges, compared to (mean ± SD) 12.16 ± 6.26 μg/m^3^ for the collocated Environment Canada chemiluminescence sampler. The Ogawa badges had an overestimation median bias compared to the NAPS method of 14%.

Comparisons between the Harvard Coarse Impactors collocated at the NAPS site with Environment Canada’s tapered element oscillating microbalances (TEOM) were performed for PM_2.5_ in both winter and summer seasons. Mean (±SD) PM_2.5_ from Harvard Coarse Impactors at the NAPS site were 8.44 ± 2.59 μg/m^3^ and 7.27 ± 1.54 μg/m^3^, whereas the TEOM measurements were 3.40 ± 0.90 μg/m^3^ and 5.63 ± 1.59 μg/m^3^, in the winter and summer, respectively. The bias was significant, with the Harvard Coarse Impactor over predicting with a median bias of 158% in winter, and 31% in summer. As reported elsewhere, the TEOM generally reads lower than filter-based methods due to its elevated inlet temperature, which causes a proportion of the volatiles in the particulates to be vaporized on intake. The volatilization may be greater in winter than in the summer, due to the greater temperature difference between the ambient air and the filter [11,12]. This could in part explain the bias seen between the two PM_2.5_ methods.

### 3.4. Predictors for Indoor Levels

Multivariate models are presented in Table 3. Models were developed separately for all homes and non-smoking homes for benzene (winter only), acetaldehyde (winter only), PM_2.5_ (both seasons) and NO_2_ (summer only). For all other pollutants, cigarette smoke was not a major contributor to indoor concentrations, and therefore only models for all homes were developed. When developing the models for all homes, the stronger predictor representing smoking between a) smoking as a dichotomous variable (yes/no), and b) the number of cigarettes smoked (as a continuous variable) was used. Due to the relatively small number of homes with smokers present, there was insufficient power to create models for smoking homes only. In the models, homes are excluded where data were missing for independent variables.

#### 3.4.1. VOC (Benzene and toluene)

Benzene models for all homes predicted 49% and 43% of the variability in indoor levels for winter and summer, respectively. For non-smoking homes in the winter, 52% of the variability was explained by the model. The presence of an attached garage was consistently associated with higher benzene levels in all models. In the summer model, having windows open decreased indoor benzene levels, while air conditioning increased indoor levels, suggesting that ventilation influences indoor concentrations, potentially due to outdoor concentrations being lower than indoors, as discussed previously.

Toluene models predicted 21% and 39% of the variability in indoor levels in the winter and summer seasons, respectively. In the summer model, increased levels of toluene indoors were found to result from attached garages, the presence of new furniture or rugs, and the use of air conditioning, whilst open windows resulted in a reduction in levels. In the winter, the only predictor was the building age, with higher levels in newer homes. The differences in predictors between summer and winter seasons is likely a result of having a short measurement period (24-h) and only partial overlap in homes measured across each season.

In our study, average indoor relative humidity was associated with decreased levels for both toluene and benzene in the summer season. As well, air conditioning was associated with higher levels of these pollutants. Since the use of AC generally tends to lead to dryer indoor air, the interaction between these two variables could explain the association observed with relative humidity. However, in our models, relative humidity was not statistically different between homes with and without AC. Use of AC also potentially indicates the closing of windows and doors resulting in tighter homes, which could explain the accumulation of these pollutants that have significant indoor sources.

Findings from studies conducted in a comparable climate and housing stock suggest a range in average indoor air concentrations of 1.2 to 5.4 μg/m^3^ for benzene and 4.7 to 41.5 μg/m^3^ for toluene [6,13–16]. Our results are within these values for both pollutants. Consistent with results from previous studies, benzene and toluene were highly influenced by indoor sources such as the presence of an attached garage, and recent renovations or addition of furniture [6,14,17–19].

#### 3.4.2. Formaldehyde

For formaldehyde, in winter and summer seasons, respectively, there were 3% and 16% of the homes that were above Health Canada’s long-term exposure limit (as measured over at least 8 h) of 50 μg/m^3^ [20]. For the winter season, this is slightly lower than what was found in a study conducted by Health Canada in Quebec City [21], where 11% of the homes were above that same guideline. Long-term exposure to concentrations above this level may result in increased respiratory symptoms such as coughing and wheezing, and allergic sensitivity, especially in children [20].

Models for formaldehyde predicted 49% of the variability in indoor levels in winter, and 36% in the summer. The year of construction of the home was consistently associated with indoor levels, with newer homes having higher concentrations. Ventilation parameters were also important predictors, and included the use of air conditioning (increasing levels in summer), open windows (decreasing levels in summer) and air exchange rate (decreasing levels with higher air exchange rate in winter). Temperature and relative humidity also increased formaldehyde levels in the winter. The higher levels of formaldehyde seen with the presence of a kitchen fan venting to the outdoors may be due to the fact that a negative pressure was created, leading to accelerated off-gassing indoors; however, more research is needed to explain this association.

Formaldehyde levels measured in this study are comparable to levels measured recently in other cities with similar climate and housing stock, where average levels ranged from 25.5 to 39.0 μg/m^3^ [20,22–25]. In a recent study by Hun *et al.* (2010) conducted in different parts of the United States, formaldehyde levels measured in homes over different seasons were considerably lower (mean = 20.9 μg/m^3^) than in our sample; however, their air exchange rates were much higher (mean ± SD = 1.07 ± 0.82 ACH) [26]. The different climatic conditions and building construction may also explain the difference in air exchange rates, and therefore in the formaldehyde levels measured between the two studies. In accordance with previous studies [20,22–25,27–29], results from our study suggest that off-gassing is a more important source of formaldehyde than combustion.

#### 3.4.3. Acetaldehyde

Acetaldehyde models in summer explained 32% of the indoor concentrations in all homes by including open windows (decreasing levels) and the use of air conditioning (increasing levels), suggesting the presence of indoor sources. In winter, decreasing levels were seen with increasing air exchange rates, and elevated concentrations were found with increasing relative humidity. The use of the stove for cooking with oil was also associated with elevated concentrations. Models for the winter season predicted 32% (non-smoking homes) and 29% (all homes) of the variability for indoor levels.

Acetaldehyde levels in Regina were lower than what was measured in other studies, where average concentrations ranged from 18.1 to 39.6 μg/m^3^ [6,22,24,25]. Acetaldehyde in our models was strongly associated with several ventilation parameters, as well as with cooking with oil and cigarette smoke (winter models only). In a study in commercial kitchens in Hong Kong, cooking with oil was associated with higher levels of carbonyls such as formaldehyde, acetaldehyde, and acrolein [30]. As opposed to formaldehyde, our study suggests that acetaldehyde levels in indoor air are more influenced by combustion than off-gassing processes.

#### 3.4.4. Fine particulate matter (PM_2.5_)

For PM_2.5_, there were 4% and 5% of homes in winter and summer, respectively that were above Health Canada’s acceptable long-term exposure guideline of 40 μg/m^3^ (minimum sampling period of 24 h) [7]. All homes above that level were homes that reported smoking during sampling. This guideline is currently being revised by Health Canada to take into account new science.

Even with the inclusion of homes with smokers, indoor PM_2.5_ levels in our study sample were still on the lower end of what has been measured elsewhere in single-family homes, where average levels ranged from 6.7 to 32 μg/m^3^ [31–45]. This could be due in part to the relatively low levels of outdoor PM_2.5_ in Regina.

Cigarette smoke was an important predictor for PM_2.5_, especially in the summer, as models including smokers predicted 53% (summer) and 37% (winter), whereas models predicted only 11% (summer) and 37% (winter) of the variability of levels in non-smoking homes. Outdoor particulate matter was associated with increased indoor levels in all models apart from summer non-smoking homes. Interestingly, the use of a barbecue outside of the house increased indoor levels in summer models. Measures of ventilation such as having a vented kitchen fan (winter models for all homes and non-smoking homes) and having a premium filter on the furnace (summer non-smoking homes) decreased indoor PM_2.5_ levels. The use of candles increased levels in the winter non-smoking model.

Activities such as smoking and burning candles are known sources of indoor PM_2.5_ [46,47]. Furthermore, ambient particles penetrate indoors very efficiently by infiltration through the building structure, cracks and opened windows, and can lead to increased indoor levels [48,49]. This was also the case in our study for PM_2.5_ emissions from the use of a barbecue outdoors in the summer [50]. Of interest, practices and technologies aimed at reducing indoor particulate exposure levels, such as the use of a premium filter on the furnace and a vented kitchen fan, seemed to have the expected effect of reducing concentrations in our study sample. This is a relatively new area of research, and only a few studies have characterized the impact of these measures on indoor levels [51–53].

#### 3.4.5. Nitrogen Dioxide (NO_2_)

All homes were below Health Canada’s acceptable long-term exposure guideline value for NO_2_ of 100 μg/m^3^ (minimum sampling period of 24 h) [7]. This guideline is currently being revised by Health Canada to take into account new science.

A limited number of studies have measured indoor NO_2_ concentrations in Canada. Gilbert *et al.* [20] measured indoor concentrations of NO_2_ in residences in Quebec City that ranged from 3.3 to 29.1 μg/m^3^, with a mean of 9.5 μg/m^3^. As well, three monitoring surveys conducted in communities in Alberta have measured indoor median concentrations ranging from 5.5 to 14.3 μg/m^3^, and 95^th^ percentile values ranging from 17.7 to 47.5 μg/m^3^ [33,54,55]. Concentrations measured in our study seem to be in agreement with these other Canadian studies; however, the maximum concentration in Regina was considerably higher, reaching 84.3 μg/m^3^. It is of note that about one-third of the homeowners in our summer sample had gas stoves, while cooking with gas is not prevalent in Canada. In fact, the *2007 Survey of Household Energy Use* conducted by the Department of Natural Resources reported that about 5% of homes across Canada use natural gas as the main energy source for their regular stove [56]. This suggests that our study homes are not necessarily typical of Canadian homes in this respect.

Cigarette smoke was an important predictor for NO_2_, as the inclusion of smoking homes in the summer model improved its prediction from 36% to 44%. The use of a gas stove was a strong predictor of increased NO_2_ levels indoors. NO_2_ levels outdoors positively influenced indoor levels, as did having windows open, even if outdoor levels were lower than indoors for most homes. The indoor predictors from our models for NO_2_, such as gas stoves and smoking, are consistent with what has been reported previously in the literature [20,57,58]. Predictors for increased indoor levels also included outdoor levels and windows open, which is consistent with findings from several studies investigating the influence on indoor levels of outdoor levels of NO_2_ and proxies such as proximity to traffic and major roads [57,59].

### 3.5. Design Considerations

Our study had several limitations. Selection bias cannot be ruled out. Due to potential liability issues, the recruitment was limited to homeowner-occupied premises, and therefore the results from this study cannot be directly extrapolated to the general population. We were unable to recruit all of the winter homes for participation again in the summer monitoring period. There were therefore 71 homes with measurements conducted in both seasons, and 74 homes with only one measurement, in either summer or winter. This limitation means that there were homes with different characteristics, which makes it challenging to compare season-to-season models.

Furthermore, our study measured indoor and outdoor concentrations of air pollutants for one 24-h period per season only (except for PM_2.5_ which was measured for one integrated five-day period per season), which is not necessarily representative of long-term exposure. It also does not address daily variability in concentrations, and possible differences between weekday and weekend exposures. As well, the relatively low fit (r^2^) of some of the models may be due to having only one measurement per home per season, resulting in less variability in concentrations and a lower frequency of predictive events that could explain the exposures. Finally, it was not possible to measure air exchange rate with the PFT method in the summer sampling, which limited the analysis and may have influenced the performance of some of the summer models.

## 4. Conclusions

Indoor concentrations of a wide range of air pollutants were measured as part of a large indoor air quality study in both summer and winter in Regina, Saskatchewan, Canada. The characteristics of houses and occupants’ activities investigated in this study explained between 11% and 53% of the variability in indoor air pollutant concentrations. Results from our study are generally in accordance with other indoor exposure studies recently conducted in regions with a similar climate. The results of this study provide important information on the distribution of exposure to air pollutants in residential settings and inform advice to be given to the public about how to reduce exposure to air pollutants in their homes through ventilation and other household practices.

## Figures and Tables

**Table 1.** Main housing characteristics and occupant’s activities influencing indoor air pollutant levels.

	**Summer**		**Winter**	

	**Non-smoking Homes (N=96)**	**All Homes (N=111)**	**Non-smoking Homes (N=85)**	**All Homes (N=106)**

Home year of construction ≤ 1953	13 (14%)	18 (16%)	13 (15%)	18 (17%)
1954–1963	29 (30%)	32 (29%)	26 (31%)	31 (29%)
1964–1977	15 (16%)	19 (17%)	17 (20%)	22 (21%)
1978–1999	30 (31%)	33 (30%)	22 (26%)	28 (26%)
≥ 2000	9 (9%)	9 (8%)	6 (7%)	6 (6%)

New furniture or rugs within the last year	34 (35%)	39 (35%)	34 (40%)	39 (37%)

Garage attached to home	49 (51%)	55 (50%)	32 (38%)	43 (41%)

Premium filter on furnace	35 (36%)	42 (38%)	26 (31%)	29 (27%)

Air conditioner used	47 (49%)	53 (48%)	-	-

Outdoor vented kitchen fan	31 (32%)	35 (32%)	26 (31%)	32 (30%)
Gas stove	33 (34%)	36 (32%)	5 (6%)	7 (7%)

Cooked with oil (fry, grill, sauté, or broil)	34 (35%)	37 (33%)	35 (41%)	44 (42%)

BBQ used	14 (15%)	16 (14%)	-	-

Windows open	65 (68%)	78 (70%)	12 (14%)	20 (19%)

Candles burned	4 (4%)	6 (5%)	5 (6%)	9 (8%)

Perfume, cologne, or aftershave used	32 (33%)	39 (35%)	41 (48%)	54 (51%)

Hair spray used	38 (40%)	42 (38%)	37 (44%)	43 (41%)

**Table 2.** Indoor and outdoor air pollutants for both seasons for all homes and non-smoking homes in Regina.

		**Summer**							**Winter**						

		N	Min	Max	Mean	Std^a^	Geo.^b^ Mean	Geo.^b^ Std.	N	Min	Max	Mean	Std^a^	Geo.^b^ Mean	Geo.^b^ Std.

Benzene (μg/m^3^)	All homes	105	0.18	32.33	2.72	5.03	1.28	2.99	105	0.53	17.87	2.06	2.73	1.44	2.08
	Non-smoking	91	0.18	32.33	2.66	5.22	1.19	3.01	84	0.53	17.87	1.85	2.67	1.31	1.99
	Outside	108	0.07	6.04	0.34	0.57	0.26	1.85	94	0.32	2.75	0.75	0.48	0.66	1.58

Toluene (μg/m^3^)	All homes	105	0.77	314.8	23.54	44.48	11.26	2.97	105	0.02	625.2	21.33	76.85	8.40	3.01
	Non-smoking	91	0.77	314.8	24.76	46.98	11.62	3.02	84	0.02	497.5	16.40	54.18	7.89	2.92
	Outside	108	0.17	61.93	1.94	6.01	1.01	2.47	94	0.23	14.82	1.66	2.25	1.08	2.27

Acetaldehyde (μg/m^3^)	All homes	111	0.49	48.80	12.69	8.63	10.10	2.09	104	0.49	94.20	12.58	11.05	9.74	2.11
	Non-smoking	96	0.49	48.80	12.44	8.64	9.85	2.12	83	0.49	32.15	10.68	6.37	8.70	2.05

Formaldehyde (μg/m^3^)	All homes	111	5.09	93.88	35.46	17.93	31.08	1.72	104	7.46	72.48	25.73	11.52	23.39	1.56
	Non-smoking	96	5.09	93.88	36.82	18.60	32.12	1.75	83	7.46	72.48	26.33	12.12	23.83	1.58

Acrolein (μg/m^3^)	All homes	111	0.44	6.81	1.35	0.99	1.05	2.04	104	0.46	3.26	0.69	0.54	0.60	1.58
	Non-smoking	96	0.44	3.89	1.24	0.80	1.00	1.97	83	0.46	3.23	0.59	0.38	0.54	1.39

PM_2.5_ (μg/m^3^)	All homes	105	1.00	82.09	9.51	12.21	6.43	2.21	95	0.88	92.12	9.42	13.46	5.46	2.61
	Non-smoking	91	1.00	24.92	6.18	3.56	5.32	1.75	76	0.88	27.61	5.47	4.95	4.13	2.05
	Outside	102	2.22	14.43	7.67	2.68	7.25	1.40	95	0.20	10.81	6.68	1.99	6.24	1.58

Elemental carbon (μg/m^3^)	All homes	51	0.20	0.74	0.25	0.09	0.24	1.26							
	Non-smoking	46	0.20	0.49	0.24	0.05	0.24	1.18							
	Outside	45	0.20	0.57	0.27	0.09	0.26	1.30							

NO_2_ (μg/m^3^)	All homes	110	1.52	84.32	11.32	10.97	8.51	2.09							
	Non-smoking	96	1.52	53.80	10.28	8.22	8.00	2.05							
	Outside	111	0.16	32.04	6.90	4.76	5.43	2.16							

O_3_ (μg/m^3^)	All homes	110	0.00	9.03	1.34	1.54	0.12	51.55							
	Non-smoking	96	0.00	9.03	1.31	1.58	0.09	60.80							
	Outside	110	5.24	16.60	10.57	2.59	10.25	1.29							

a Standard deviation

b geometric statistics.

**Table 3.** Determinants of indoor air pollutant concentrations in Regina.

**Summer**				**Winter**			

**Independent variable**	**Estimate (β)**	**Standard error**	**p-value**	**Independent variable**	**Estimate**	**Standard error**	**p-value**
**Benzene (Natural logarithm of concentrations in μg/m^3^)**							
*All homes N = 101*			*R^2^ = 0.43*	*All homes N = 91*			*R^2^ = 0.49*
Intercept	0.666	0.554	0.232	Intercept	−0.386	0.125	0.003
Average indoor RH	−0.021	0.012	0.089	Outdoor benzene	0.468	0.120	<0.001
Air conditioning used	0.408	0.183	0.028	Attached garage	0.860	0.116	<0.001
Windows open	−0.393	0.201	0.053	Smoking inside	0.709	0.160	<0.001
Attached garage	1.058	0.177	<.001				
				
				*Non-smoking homes N = 75*			*R^2^ = 0.52*
				Intercept	−0.437	0.118	<0.001
				Outdoor benzene	0.371	0.114	0.002
				Attached garage	0.852	0.120	<0.001
				Hair spray used	0.301	0.122	0.016

**Toluene (Natural logarithm of concentrations in μg/m^3^)**							

*All homes N = 101*			*R^2^ = 0.39*	*All homes N = 104*			*R^2^ = 0.21*
Intercept	3.181	0.580	<0.001	Intercept	2.523	0.414	<0.001
New furniture or rugs^a^	0.513	0.183	0.006	Construction year (reference group = ≥ 2000)			<0.001^b^
Attached garage	0.704	0.184	<0.001	≤ 1953	−1.014	0.478	0.036
Windows open	−0.589	0.209	0.006	1954–1963	−0.472	0.452	0.298
Average indoor RH	−0.026	0.013	0.041	1964–1977	−0.718	0.467	0.127
Air conditioning used	0.375	0.192	0.054	1978–1999	0.309	0.457	0.501

**Formaldehyde (Natural logarithm of concentrations in μg/m^3^)**							

*All homes N = 107*			*R^2^ = 0.36*	*All homes N = 72*			*R^2^ = 0.49*
Intercept				Intercept	1.677	0.438	<0.001
Air conditioning used	0.245	0.094	0.011	Average indoor RH	0.020	0.005	<0.001
Open windows	−0.326	0.103	0.002	Average temperature	0.065	0.017	<0.001
Vented kitchen fan	0.199	0.097	0.043	Air exchanges/hour	−0.237	0.106	0.030
Construction year (reference group = ≥ 2000)			0.007^b^	Construction year (reference group = ≥ 2000)			0.001^b^
≤ 1953	−0.099	0.200	0.621	≤ 1953	−0.500	0.193	0.012
1954–1963	−0.436	0.184	0.020	1954–1963	−0.415	0.170	0.017
1964–1977	−0.150	0.201	0.459	1964–1977	−0.223	0.173	0.202
1978–1999	−0.028	0.182	0.877	1978–1999	−0.037	0.167	0.826

**Acetaldehyde (Natural logarithm of concentrations in μg/m^3^)**							

*All homes N = 107*			*R^2^ = 0.32*	*All homes N = 72*			*R^2^ = 0.29*
Intercept	2.205	0.154	<0.001	Intercept	1.554	0.275	<0.001
Windows open	−0.377	0.140	0.008	Air exchanges/hour	−0.377	0.199	0.063
Air conditioning used	0.597	0.128	<0.001	Average indoor RH	0.029	0.010	0.006
Perfume used^b^	0.249	0.126	0.051	Cooked with oil	0.299	0.165	0.074
				Smoking inside	0.661	0.224	0.004
				
				*Non-smoking homes*	*N = 58*		*R^2^ = 0.32*
				Intercept	1.625	0.347	<0.001
				Air exchanges/hour	−0.830	0.377	0.032
				Average indoor RH	0.032	0.012	0.007
				Cooked with oil	0.289	0.171	0.096

**PM_2.5_ (Natural logarithm of concentrations in μg/m^3^)**							

*All homes N = 101*			*R^2^ = 0.53*	*All homes N = 87*			*R^2^ = 0.37*
Intercept	1.253	0.174	<0.001	Intercept	1.012	0.310	0.002
PM_2.5_ outdoors	0.036	0.021	0.086	PM_2.5_ outdoors	0.111	0.044	0.014
BBQ used	0.284	0.109	0.011	Vented kitchen fan	−0.593	0.185	0.002
Number of cig. smoked	0.022	0.002	<0.001	Number of cig. smoked	0.017	0.003	<0.001

*Non-smoking homes N = 91*			*R^2^ = 0.11*	*Non-smoking homes N = 59*			*R^2^ = 0.37*
Intercept	1.606	0.088	<0.001	Intercept	0.147	0.353	0.679
BBQ used	0.304	0.112	0.008	PM_2.5_ outdoors (μg/m^3^)	0.076	0.040	0.061
Premium furnace filter	−0.255	0.118	0.034	Vented kitchen fan	−0.267	0.153	0.088
				Average indoor RH	0.034	0.009	<0.001
				Candles used	0.486	0.172	0.007

**NO_2_ (Natural logarithm of concentrations in μg/m^3^)**							

*All homes N = 109*			*R^2^ = 0.44*				
Intercept	1.367	0.125	<0.001				
NO_2_ outdoors	0.031	0.011	0.007				
Gas stove	0.836	0.115	<0.001				
Smoking inside	0.487	0.169	0.005				
Windows open	0.307	0.120	0.012				
				
*Non-smoking homes N=95*			*R^2^ = 0.36*				
Intercept	1.399	0.141	<0.001				
NO_2_ outdoors (ppb)	0.029	0.014	0.044				
Gas stove	0.793	0.125	<0.001				
Windows open	0.298	0.127	0.025				

a within the past year

b overall p-value for categorical variable

c Use of perfume, cologne or aftershave.
